# Toward Sustainable Composites: Graphene‐Modified Jute Fiber Composites with Bio‐Based Epoxy Resin

**DOI:** 10.1002/gch2.202300111

**Published:** 2023-08-14

**Authors:** Mohammad Hamidul Islam, Shaila Afroj, Nazmul Karim

**Affiliations:** ^1^ Centre for Print Research The University of the West of England Bristol BS16 1QY UK

**Keywords:** bio‐epoxy resins, graphene, natural fibers, sustainable composites

## Abstract

Sustainable natural fiber reinforced composites have attracted significant interest due to the growing environmental concerns with conventional synthetic fiber as well as petroleum‐based resins. One promising approach to reducing the large carbon footprint of petroleum‐based resins is the use of bio‐based thermoset resins. However, current fiber‐reinforced bio‐based epoxy composites exhibit relatively lower mechanical properties such as tensile, flexural strength, and modulus, which limits their wider application. Here the fabrication of high‐performance composites using jute fibers is reported, modified with graphene nanoplates (GNP) and graphene oxide (GO), and reinforced with bio‐based epoxy resin. It is demonstrated that physical and chemical treatments of jute fibers significantly improve their fiber volume fraction (*V_f_*) and matrix adhesion, leading to enhanced mechanical properties of the resulting Jute/Bio‐epoxy (J/BE) composites. Furthermore, the incorporation of GNP and GO further increases the tensile and flexural strength of the J/BE composites. The study reveals the potential of graphene‐based jute fiber‐reinforced composites with bio‐based epoxy resin as a sustainable and high‐performance material for a wide range of applications. This work contributes to the development of sustainable composites that have the potential to reduce the negative environmental impact of conventional materials while also offering improved mechanical properties.

## Graphical abstract

The fabrication of high‐performance sustainable composites using jute fibers modified with graphene‐based materials and reinforced with bio‐based epoxy resin is reported. The study reveals the potential of graphene‐based jute fiber‐reinforced composites with bio‐based epoxy resin as a sustainable and high‐performance material for a wide range of applications.

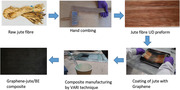

## 1 Introduction

In recent years, there has been growing concern over the environmental impact of synthetic materials, which has led to increased interest in sustainable and biodegradable materials.^[^ ^1^, ^2^, ^3^ ^]^ Natural fibers, such as jute, have become an attractive option for reinforcing bio‐based composites due to their sustainability and environmental benefits. Jute is a plant fiber that captures carbon and produces oxygen during cultivation and can be recycled and biodegraded. Additionally, jute fibers require fewer chemicals during processing, making them an environmentally friendly alternative to synthetic fibers. Therefore, jute fiber reinforced polymer (JFRP) composites have gained significant attention in recent years due to their low environmental impact, biodegradability, and low cost compared to synthetic fiber reinforced polymer (SFRP) composites.^[^ ^4^, ^5^, ^6^, ^7^, ^8^, ^9^, ^10^ ^]^

Despite their many benefits, jute fibers can suffer from poor mechanical properties and weak adhesion when reinforced with a matrix due to the presence of large amounts (20 wt.%–50 wt.%) of noncellulosic materials such as hemicellulose and lignin.^[^ ^11^ ^]^ Such materials reduce the crystallinity and hydrophilicity of the fibers, which ultimately results in poor mechanical properties of the composites.^[^ ^12^ ^]^ However, by improving the interfacial bonding between the fiber and matrix, it is possible to enhance the mechanical and interfacial properties of the composites. Surface treatment and modification of jute fibers are therefore considered essential for improving their adhesion to a polymer matrix.^[^ ^13^ ^]^ A range of physical and chemical treatments have been investigated to remove the noncellulosic materials and impurities from the interfibrillar region of jute fibers and improve their mechanical properties. Among these treatments, hot water^[^ ^8^ ^]^ and alkali treatments are the most commonly used methods for removing noncellulosic materials and improving the performance of jute fiber and its composites.^[^ ^6^, ^14^, ^15^, ^16^ ^]^

Graphene and its derivatives, such as graphene nanoplates (GNP), graphene oxide (GO), and reduced graphene oxide (rGO), have drawn significant research interest in recent years as promising materials for manufacturing multifunctional textiles^[^ ^17^, ^18^, ^19^, ^20^ ^]^ and composites.^[^ ^21^, ^22^, ^23^, ^24^ ^]^ The enthusiasm surrounding graphene and its derivatives is fueled by their exceptional mechanical, electrical, and thermal properties.^[^ ^25^, ^26^ ^]^ The unique combination of these properties has positioned graphene's derivative as potential filler materials for high‐performance fiber‐reinforced polymer (FRP) composites applications.^[^ ^11^, ^23^, ^27^ ^]^ In particular, GO can be produced in large quantities as a stable dispersion, and its coating on fiber surfaces can improve fiber/matrix bonding and enhance the strength and toughness of composites.^[^ ^8^ ^]^ Recent studies have demonstrated that the nanosilica‐decorated GO fillers can improve the mechanical properties of the jute fiber/epoxy composites.^[^ ^28^, ^29^ ^]^ Similarly, GNP, which are composed of a few layers of graphene stacked together in a plate‐like shape, can be produced at a relatively low cost through mechanical or liquid‐phase exfoliation of pre‐treated graphite. Such materials have been extensively investigated in fiber reinforced polymer (FRP) composites for their potential to improve structural, non‐structural, and multifunctional properties.^[^ ^6^, ^8^, ^13^, ^30^, ^31^, ^32^, ^33^ ^]^ However, most researchers use fossil fuel‐based polymer matrices to manufacture natural fiber composites, which are extremely challenging to recycle and not environmentally friendly.^[^ ^10^ ^]^ Therefore, the use of bio‐based epoxy resin as a matrix for jute fiber reinforced composites, along with the incorporation of graphene and its derivatives, presents a promising avenue for the development of eco‐friendly and sustainable composites.

Bio‐based epoxy resin is a promising class of bio‐sourced resin that is synthesized from renewable precursors, such as unsaturated vegetable oils, saccharides, tannins, cardanol, terpenes, rosins, and lignin.^[^ ^34^, ^35^, ^36^ ^]^ The bio‐based content in bio‐epoxy is generally measured as the percentage of naturally occurring carbon in the materials. The US Department of Agriculture (USDA) labelling system specifies that bio‐epoxies must contain at least 25% bio‐content. Currently, a few bio‐epoxies contain a total bio‐content of ≈30%, which can significantly reduce greenhouse gas emissions.^[^ ^37^ ^]^ Compared to fossil fuel‐derived epoxy resins, bio‐based epoxy resins have lower volatile organic compound (VOC) emissions, no unpleasant odors, and reduced dependence on fossil fuels for resin processing. As a result, the global bio‐based epoxy resin market is projected to grow at a steady pace. In 2020, the global bio‐based epoxy resin market was valued at ≈US$ 4.8 billion, and it is expected to reach a value of US$ 8.6 billion by 2031^[^ ^38^ ^]^ Recently some researchers have employed bio‐epoxy resins to fabricate JFRP composites.^[^ ^39^, ^40^, ^41^ ^]^ Some of these reported composites^[^ ^39^ ^]^ have exhibited relatively low mechanical properties, such as tensile, flexural strength, and modulus. Therefore, it is crucial to develop JFRP composites reinforced with bio‐epoxy resin with improved mechanical properties.

In this study, we address the challenges of poor mechanical properties and low interfacial adhesion of jute fiber‐reinforced bio‐epoxy resin composites by exploring the use of various physical, chemical, and graphene‐modified jute fibers as reinforcements. To achieve this, we employed a vacuum‐assisted resin infusion process, which is an efficient and cost‐effective method for fabricating jute fiber reinforced bio epoxy composite. We investigated the effect of jute fiber surface modification on the mechanical properties of the resulting composites, specifically their tensile and flexural properties. We focused on physical and chemical treatments such as hot water and alkali treatment, as well as graphene‐based modifications, including GNP and GO. To evaluate the effectiveness of such modifications, we conducted a comprehensive analysis of the fracture surface of the tested specimens using scanning electron microscopy (SEM). We believe this research contributes to the growing body of knowledge on sustainable green composites and their potential for use in a range of applications, from structural and non‐structural to multifunctional materials.

## 2 Results and Discussion

To remove the non‐cellulosic materials of the raw jute fibers, hot water and alkali treatments were carried out that improved the fiber‐matrix interfacial bonding. Different types of jute fiber preforms were prepared from raw jute, and hot water and alkali treated jute fibers by a hand combing method. Hot water and alkali‐treated jute fibers were also modified with GO and GNP using a simple dip coating method to further improve the mechanical properties. Six different types of J/BE UD composites were manufactured using the vacuum‐assisted resin infusion (VARI) process. They are untreated jute/BE (UT J/BE), untreated combed jute/BE (UTC J/BE), hot water treated combed jute/BE (HWC J/BE), hot water and alkali treated combed jute/BE (HWAC J/BE), hot water and alkali treated combed and GO modified jute/BE (HWACGO J/BE) and hot water and alkali treated combed and GNP modified jute/BE (HWACGNP J/BE). The tensile and flexural properties of different jute fiber composites were studied.

### 2.1 Tensile Properties

**Figure 1:**
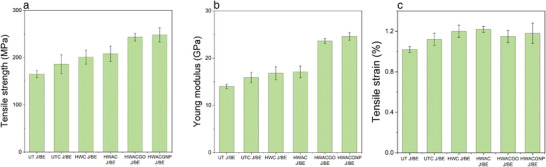
a) Tensile strength, b) Young modulus and c) Strain% of different jute/bio‐epoxy composites. Mean values and standard deviation (SD) were calculated from 5 specimens tested for each group. The error bar indicates the SD.

**Table** **1** summarizes the fiber volume fraction, density, and mechanical properties of the different J/BE composites. The mean tensile strength, Young's modulus, and strain% with standard deviation (SD) are shown in **Figure** **1**a–c. The tensile stress–strain graphs of each group of samples are presented in the supporting document (Figure S1, Supporting Information). A significant improvement in the tensile strength and modulus of the different composites was observed. This improvement can be attributed to two main reasons. The first reason is that physical and chemical treatments help to improve the fiber volume fraction (*V_f_*) and fiber‐matrix adhesion. The *V*_*f*_ has a significant influence on the mechanical properties of composites. In a UD composite, fiber‐dominated properties such as strength and stiffness increase proportionally with the increase of *V*_*f*_ up to ≈80%, at which the amount of resin is sufficient to hold the fibers properly.^[^ ^42^ ^]^

**Table 1:** Fiber volume fraction, density and mechanical properties of the different jute fiber/BE composites

Composite code	Fiber volume fraction [%]	Composite density [g/cm^3^]	Tensile properties			Flexural properties		
			Strength [MPa]	Modulus [GPa]	Strain [%]	Strength [MPa]	Modulus [GPa]	Strain [%]
UT J/BE	30.1 ± 1.3	1.22 ± 0.07	165 ± 7.4	14.0 ± 0.5	1.02 ± 0.03	145 ± 8.2	10.5 ± 0.9	2.27 ± 0.18
UTC J/BE	36.6 ± 1.2	1.22 ± 0.05	186 ± 20.1	15.9 ± 1.1	1.12 ± 0.06	167 ± 14.9	13.9 ± 1.5	2.29 ± 0.18
HWC J/BE	43 ± 1.1	1.25 ± 0.06	201 ± 14.8	16.8 ± 1.4	1.20 ± 0.07	194 ± 16.6	16.5 ± 1.4	2.13 ± 0.22
HWAC J/BE	44.8 ± 1.0	1.26 ± 0.04	208 ± 16.3	17.1 ± 1.3	1.22 ± 0.03	211 ± 10.2	18.1 ± 1.2	2.13 ± 0.16
HWACGO J/BE	46.1 ± 1.2	1.25 ± 0.06	243 ± 8.2	23.6 ± 0.6	1.15 ± 0.06	221 ± 13.2	20.7 ± 1.6	2.03 ± 0.10
HWACGNP J/BE	45.2 ± 1.1	1.23 ± 0.05	248 ± 15.1	24.6 ± 0.8	1.18 ± 0.09	223 ± 8.4	21.4 ± 1.4	2.05± 0.06

The value after ± is the standard uncertainty of the measurement.

The UT jute fiber composite had a lower *V_f_* that increased after physical treatment such as hand combing. The *V_f_* increased by ≈7% in the UTC J/BE composite. The combing process breaks down the mesh structure of the UT jute fiber bundles and separates the fibers, which brings them closer together and results in better mechanical properties. The tensile strength and modulus of the UTC J/BE increased by ≈12.7% and ≈13.6%, respectively, compared to the UT J/BE composites. This improvement is due to the enhancement of the *V_f_*, which increased their load‐bearing capacity. For further enhancement of *V_f_* and fiber‐resin adhesion in the composites, the jute fibers were treated with hot water and alkali. The tensile strength of the HWC J/BE and HWAC J/BE composites increased by ∽21.8% and ∽26.1%, respectively, and Young's modulus increased by ∽20% and ∽22.1%, respectively, compared to the UT J/BE composites. Hot water and alkali treatment remove the waxes, lignin, and hemicellulose from the jute fiber, which helps to fibrillate the jute fiber bundles during combing. Previous studies have shown that hot water and alkali treatment improves fiber‐matrix adhesion because of the removal of natural impurities, which increases the effective surface area available for contact with the wet matrix.^[^ ^11^, ^16^ ^]^

Recent studies have reported that the coating of graphene materials on chemically treated jute fibres significantly enhances the interfacial and mechanical properties of the composites.^[^ ^6^, ^8^, ^43^ ^]^ To further improve the mechanical properties of the jute/BE composites, the surfaces of hot water and alkali‐treated jute fibers were modified with GO (1 wt.%) and GNP (1 wt.%), and the HWACGO J/BE and HWACGNP J/BE composites were manufactured. The tensile strength of the HWACGO J/BE and HWACGNP J/BE composites increased by ∽16.8% and ∽19.2%, respectively, and the Young modulus increased by ∽38% and ∽43.9%, respectively, compared to the HWAC J/BE composites. However, this improvement is even more significant when compared to UT J/BE composites. Thus, the combination of physical and chemical treatments and graphene modifications significantly improved the mechanical properties. The tensile strength of the HWACGO J/BE and HWACGNP J/BE composites increased by ∽47.3% and ∽50.3%, respectively, and the Young modulus increased by ∽68.6% and ∽75.7%, respectively, compared to UT J/BE composites. This enhancement in tensile strength and modulus of the GO and GNP‐coated JFRP composites may be due to strong adhesion between the graphene material‐treated jute fibers and the matrix, which contributes to effective stress transfer from the matrix to the fibers.

### 2.2 Flexural Properties

**Figure 2:**
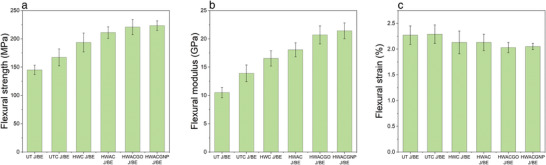
a) Flexural strength b) Flexural modulus and c) Flexural strain% of different jute/bio‐epoxy composites. Mean values and standard deviation (SD) were calculated from 5 specimens for each group. The error bar indicates the SD.

The effect of physical, chemical, and surface modifications of jute fibers on the flexural properties of different J/BE composites is shown in **Figure** **2**a–c and Table 1. The mean and SD were calculated from test results of five specimens from each group. The flexural stress–strain graphs for each group of samples are presented in the supporting document (Figure S2, Supporting Information). All physically, chemically treated, and graphene‐modified J/BE composites showed improved flexural strength and flexural modulus compared to the UT J/BE composite. The flexural strength and modulus of the HWAC J/BE composites increased by ∽45.5% and ∽72%, respectively, as compared to the UT J/BE composite due to the alkali treatment and combing of jute fibers. This improvement is due to the higher *V_f_* in the composite, as well as strong fiber‐matrix adhesion. Alkali treatment provides a rough surface topography to the jute fiber by removing the non‐cellulosic materials and improves the fiber surface adhesive characteristics.^[^ ^44^ ^]^

Moreover, GO and GNP‐modified JFRP composites exhibited higher flexural strength and modulus compared to untreated and alkali‐treated JFRP composites. The greatest improvement was observed with GNP‐modified JFRP composites. The flexural strength and modulus of HWACGNP J/BE composites were ∽5.8% and ∽18.3% higher, respectively, compared to HWAC J/BE composite and ∽54% and ∽104% higher, respectively, compared to UT J/BE composite. Similar improvements were achieved with HWACGO J/BE composite. The flexural strength and modulus of HWACGO J/BE composites were ∽4.6% and ∽14.3% higher, respectively, compared to HWAC J/BE composite and ∽52.2% and ∽97% higher, respectively, compared to UT J/BE composite. The flexural strength of FRP composites depends on the bonding between the fiber and the matrix as well as the strength of both constituents. The oxygen‐containing functional groups of GO create a link between the cellulosic fiber and matrix by forming a chemical bond with them. This chemical bond with both the cellulose and the matrix provides molecular continuity across the interface of the composite and enhances the flexural properties.

### 2.3 Fracture Surface Analysis

**Figure 3:**
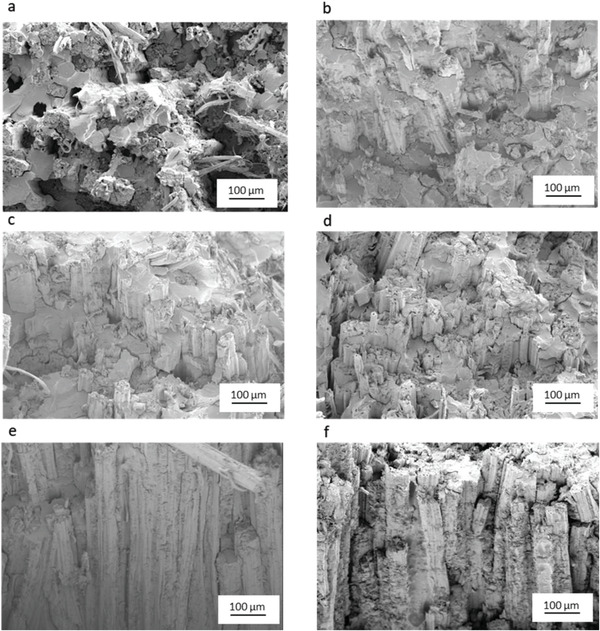
Fracture surfaces of different J/BE composites after tensile test; a) untreated J/BE, b) untreated combed J/BE, c) hot water treated combed J/BE, d) hot water and alkali treated combed J/BE, e) hot water and alkali treated combed and GO modified J/BE and f) hot water and alkali treated combed and GNP modified J/BE.

The fracture surfaces of the tensile test specimens of the composites were analyzed using SEM to evaluate the fiber‐matrix bonding. **Figure** **3**a–f shows the SEM images of different J/BE composite fracture surfaces. In the case of the UT J/BE composite without combing (Figure 3a), the fracture surface of the composites has a lot of holes, and the fiber pull‐out was observed. The fibers are in a bundle form, and some randomly oriented fibers were also observed. The holes are generally observed when the adhesion between fibers and matrix is poor.^[^ ^44^ ^]^ For this reason, when stress was applied to UT J/BE composites, the fibers were easily pulled out from the matrix. After combing the UT fibers, the fibers are partially separated and aligned, and fewer holes were observed on the fracture images (Figure 3b). The failure surfaces of HWC J/BE (Figure 3c) and HWAC J/BE (Figure 3d) composites look different from UT J/BE composites. The rate of fiber pull‐out is reduced, and the failure surface seems more uniform. These results indicate that the chemical treatment improves the interfacial bonding between the fiber and matrix. Figures 3e and f show the GO and GNP‐coated jute fiber composite fracture surfaces. Epoxy residuals on the fracture surface of HWACGO J/BE and HWACGNP J/BE composites show a relatively rougher surface compared to the without coated composite, indicating strong bonding between the resin and coated fibers. The brittle nature of the fracture surface provides proof of the strong interfacial bonding that can contribute to better mechanical properties of the composites.

### 2.4 Impact Assessment of Petroleum‐Based Epoxy Resin and Bio‐Based Epoxy Resin

**Figure 4:**
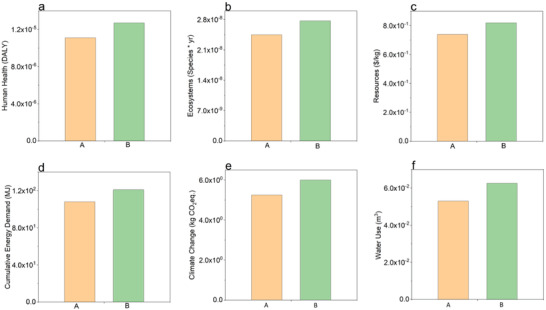
Potential cradle‐to‐gate environmental impacts analysis associated with 1 Kg of bio‐based liquid epoxy resin produced by Wessex Resins and 1 Kg industry average petrochemical‐based liquid epoxy resin products: a) Human Health, b) Ecosystems, c) Resources, d) Climate Change, e) Cumulative Energy and f) Water Use.

Most researchers use either petroleum‐based non‐biodegradable or bio‐based non‐biodegradable polymer matrices to manufacture natural fiber composites.^[^ ^45^ ^]^ To address the environmental problems caused by non‐biodegradable materials, there has been significant interest in the development of bio‐composites with improved mechanical performance. A comparative life cycle assessment (LCA)^[^ ^46^ ^]^ was carried out to evaluate the cradle‐to‐gate environmental impacts of a bio‐based liquid epoxy resin (LER) formulation produced by Wessex Resins against industry‐average petrochemical‐based LER products.^[^ ^47^ ^]^ The study investigated six impact categories: Human Health, Ecosystems, Resources, Climate Change, Cumulative Energy Demand, and Water Use. The results, presented in **Figure** **4**a–f, indicate that the bio‐based LER product has 10%–15% fewer environmental impacts and uses less energy compared to the industry‐average petrochemical‐based LER product across all impact categories.

## 3 Conclusion

This study investigated the mechanical properties and fabrication of environmentally sustainable composites made from jute and bio‐epoxy, modified with graphene derivatives. Physical and chemical treatment of the jute fibers improved the *V_f_* and adhesion between the fibers and matrix in the composites. The incorporation of GO and GNP further enhanced the tensile and flexural properties of the composites, with the HWACGNP J/BE composites showing the highest improvement of tensile (tensile strength 248 ± 15.1 MPa and modulus 24.6 ± 0.8 GPa) and flexural (flexural strength 223 ± 8.4 MPa and modulus 21.4 ± 1.4 GPa) properties. Compared to untreated J/BE composites, HWACGNP J/BE composites had an increase in tensile strength and Young's modulus by ≈50.3% and 75.7%, and flexural strength and modulus by ≈54% and 104%, respectively. Overall, these graphene‐based J/BE composites have the potential to reduce non‐biodegradable plastic waste and improve the carbon footprint of composite industries.

## 4 Experimental Section

### Materials

Tossa white jute fiber (Corchorus Olitorious) was generously donated by the Bangladesh Jute Research Institute (BJRI). The untreated long jute fiber has a golden hue, with an average length and diameter of ≈2.9 m and 0.059 mm, respectively. High bio‐based epoxy laminating resin and a slow hardener were obtained from Epoxy Revolution (Entropy Resins ONE, a USDA Certified BioPreferred® Product with 30% biobased content) in the UK, while Araldite 2011 A/B epoxy adhesive was purchased from Huntsman (USA). Analytical grade sodium hydroxide (NaOH) pellets were obtained from Scientific Laboratory Supplies Ltd., UK, and 2‐Propanol (≥99.5%) was purchased from Sigma–Aldrich, UK. GO was purchased from the Sixth Element Materials Technology Co. Ltd. (China). Graphene nanoplatelets (GNP) (xGNP, Grade M‐15, XG Science, USA) with a nominal lateral size of ∽15 µm as provided by the supplier were utilized. The manufacturer stated that the average thickness of all the flakes was approximately in the range of 6–8 nm.

### Hot Water and Alkali Treatment of Jute Fiber

A significant amount of non‐cellulosic materials, such as wax, hemicellulose, and lignin, were present in jute fibers, which weaken the fiber‐matrix interfacial bonding. Therefore, surface modification of jute fibers was required. Following a previous study,^[^ ^11^ ^]^ hot water and alkali treatments were carried out to improve the fiber‐matrix interfacial bonding. The jute fibers, cut into ∽35 cm long pieces, were dried in an oven at 80 °C to achieve a constant weight. Then, the fibers were treated with warm water at 60 °C for 60 min, followed by treatment at 100 °C for 60 min. The weight of the fibers was reduced by ≈4.5 wt.%. The hot water‐treated jute fibers were then treated with a 0.5% NaOH solution at 30 °C with a material to liquor ratio (M:L) of 1:30. The fibers were immersed in the alkali solution for 24 h, and then washed with fresh water several times to remove the remaining NaOH adhering to the surface of the fiber. Finally, the fibers were washed with distilled water. Two cycles of alkali treatment were performed using the same process.

### Preparation of Jute Fiber Preforms

**Figure 5:**
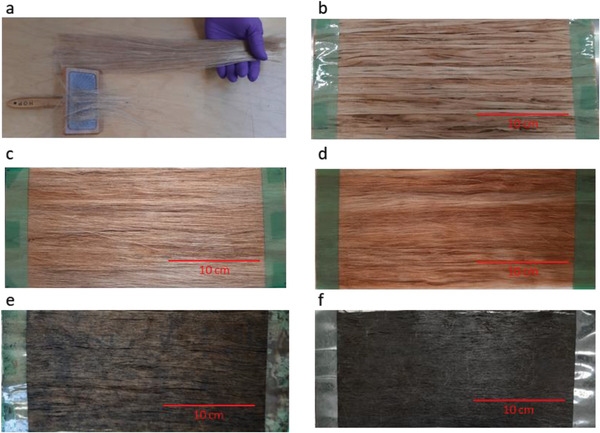
a) Hand combing process of jute fiber, b) untreated jute UD preform, c) untreated combed jute UD preform, d) hot water and alkali treated combed jute UD preform, e) hot water and alkali treated combed jute UD preform coated with GO and f) hot water and alkali treated combed jute UD preform coated with GNP.

Four types of unidirectional (UD) jute fiber preforms were prepared: untreated raw jute as received, untreated raw jute with hand combing, hot water‐treated jute with hand combing, and hot water and alkali‐treated jute with hand combing. The hand combing process was used for the individualization and parallelization of the jute fiber. The hand comb was dragged along the length of the fiber 3–4 times. Both edges of the perfectly aligned jute fiber web were sealed using double‐sided tape to prepare the UD preform that was used to manufacture the composite. The hot water and alkali‐treated jute fiber UD preform was also used for graphene modification. The dimensions of all preforms were 30 cm x 12 cm. Photographs of the combing process and UD preforms were shown in **Figure** **5**a–f.

### Preparation of GO and GNP Dispersion

GO and GNP dispersions were prepared using a bath‐type sonication method. First, the GO was mixed with deionized (DI) water and stirred with a magnetic stirrer for 2 h. Then, the GO dispersion was sonicated for 2 h to prepare a homogeneous dispersion. The GNP was not fully dispersed in water, so to prepare a perfectly homogeneous dispersion, the GNP was dispersed in 2‐Propanol (IPA) and DI water (50% propanol + 50% water) using a similar process as the GO dispersion. For both GO and GNP, 1% wt. (wt./wt.) dispersions were prepared.

### Coating of the Jute Fiber Preform

Preforms made from hot water and alkali‐treated jute fibers were coated with GO and GNP using a dip coating method. The performers were immersed in GO and GNP dispersions for 30 min and subsequently air dried overnight, followed by drying at 60 °C for 1 h. The coated jute fiber preforms were shown in Figure 5e,f.

### Fabrication of Composite Laminates Using UD Preforms

Six different types of jute/bio‐epoxy UD composites were manufactured using the VARI process. Three layers of UD preforms were laid on a metal plate that was precoated with a release agent to ensure easy de‐molding of composites. A peel ply was used on the top side of the layered UD preform. Additionally, a mesh fabric was placed on top to ensure an even flow of resin during the infusion process. The preforms were sealed with a plastic bag and vacuum‐pressed using a pump. Bio‐epoxy laminating resin and slow hardener were degassed separately for 1 h and mixed. The mixed resin was again degassed for 10 min to ensure that there were no bubbles inside the resin. Finally, the resin was carefully sucked into the preform through the resin inlet and outlet tube using a vacuum pump. The resin‐infused preforms were cured at room temperature for 48 h. The composite manufacturing flow diagram was shown in Figure S3 (Supporting Information). The list of different laminates with their codes was presented in **Table** **2**.

**Table 2:** List of different jute/bio‐epoxy composite laminates

Panel no.	Preform type	Composite code
1	Untreated jute	UT J/BE
2	Untreated combed jute	UTC J/BE
3	Hot water treated combed jute	HWC J/BE
4	Hot water and alkali treated combed jute	HWAC J/BE
5	Hot water and alkali treated combed and GO modified jute	HWACGO J/BE
6	Hot water and alkali treated combed and GNP modified jute	HWACGNP J/BE

### Tensile Strength Testing of Composites

Tensile tests were carried out on the composites in accordance with ASTM D3039M standard, using a Testometric X350‐20 testing machine (UK) equipped with a 20 kN load cell at a crosshead speed of 2 mm min^−1^. The strain was measured using a mechanical extensometer with a nominal gauge length of 25 mm. Five specimens (250 mm long and 15 mm wide) were prepared for each type of composite. End tabs made of glass fiber‐reinforced cross‐ply plates with a thickness of 1.60 mm were bonded to the specimens using a two‐component Araldite 2011 A/B epoxy adhesive.

### Flexural Test

Flexural tests were carried out using a Testometric X350‐20 (UK) testing machine equipped with a 20 kN load cell and a crosshead speed of 1 mm min^−1^, in accordance with the ASTM D‐790 standard for a span‐to‐depth ratio of 32:1. At least five specimens were tested for each type of composites.

### Statistical Analysis

Five specimens were tested for each group of samples for tensile and flexural tests. The mean and standard deviations (SD) were calculated using the Excel software. The values presented in the table were mean ± SD.

### Characterization

The surface topography of the fractured specimens was analyzed using an FEI Quanta 650 Field Emission Scanning Electron Microscope (FESEM). To avoid charging, all the specimens were gold‐coated using an Emscope SC500 gold sputter coating unit before observation.

## Conflict of Interest

The authors declare no conflict of interest.

## Supporting information

Supporting Information

## Data Availability

The data that support the findings of this study are available from the corresponding author upon reasonable request.
