# Supplementary material for: Toward Sustainable Composites: Graphene‐Modified Jute Fiber Composites with Bio‐Based Epoxy Resin
Source: Glob Chall. 2023 Aug 14;7(9):2300111. doi: 10.1002/gch2.202300111 (PMC10517308; doi:10.1002/gch2.202300111)
Supplement: Supplementary file 1 — Supporting Information [file GCH2-7-2300111-s001.pdf]

Global Challenges
Open Access

Supporting Information

for *Global Challenges.*, DOI 10.1002/gch2.202300111

Toward Sustainable Composites: Graphene-Modified Jute Fiber Composites with Bio-Based Epoxy Resin

*Mohammad Hamidul Islam, Shaila Afroj and Nazmul Karim**

Supporting Information

Towards Sustainable Composites: Graphene-Modified Jute Fibre Composites with Bio-Based Epoxy Resin

*Mohammad Hamidul Islam, Shaila Afroj, and Nazmul Karim**

Dr. M. H. Islam, Dr. S. Afroj, Dr. N. Karim

Centre for Print Research, The University of the West of England, Bristol, BS16 1QY, UK

E-mail: nazmul.karim@uwe.ac.uk

Supporting Information 1: Tensile properties of different jute/bio-epoxy composites

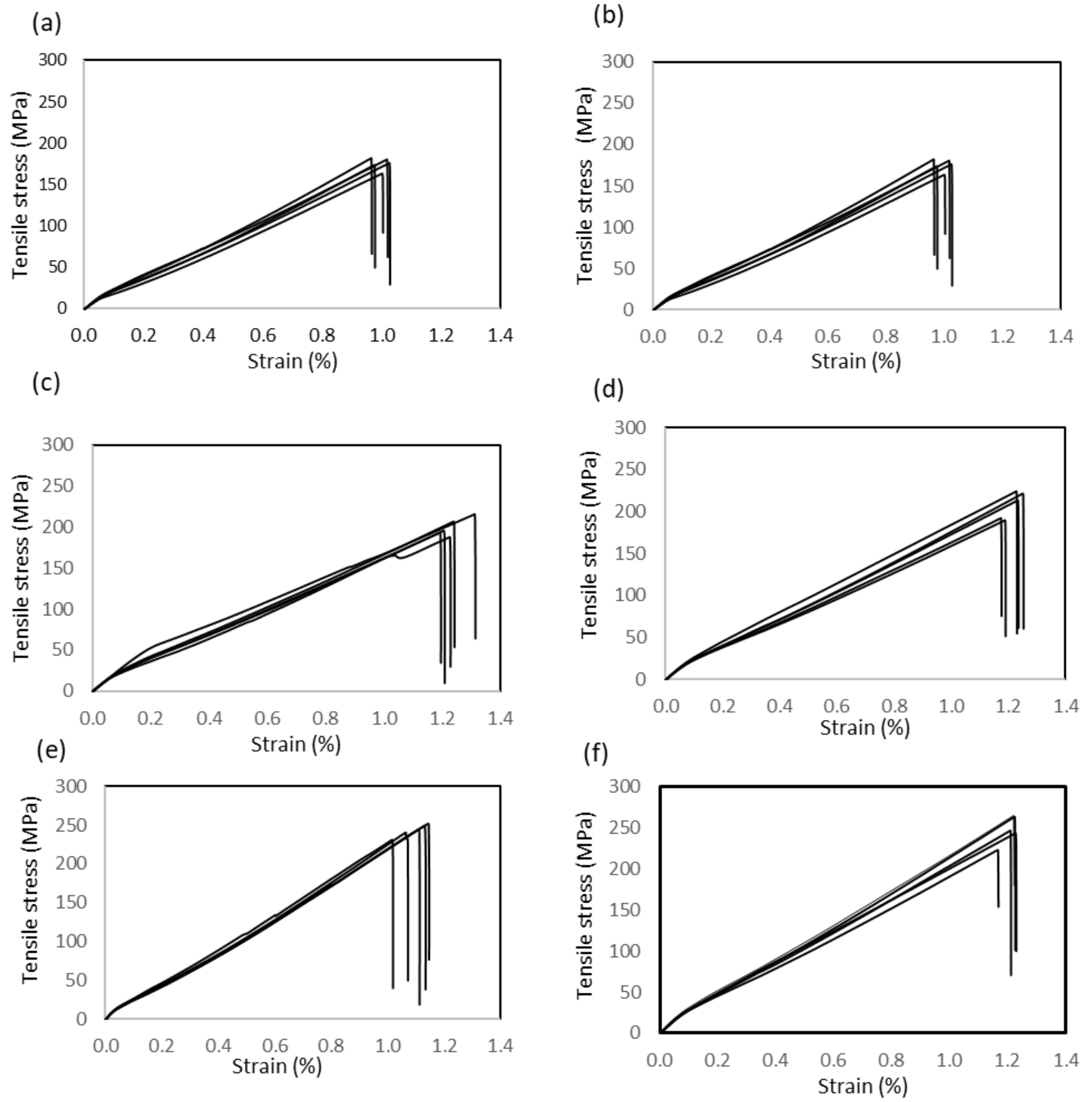

Figure S1: Tensile stress-strain graph of different jute/bio-epoxy (BE) composites; (a) untreated jute/BE (UT J/BE), (b) untreated combed jute/BE (UTC J/BE), (c) hot water treated combed jute/BE (HWC J/BE), (d) hot water and alkali treated combed jute/BE (HWAC J/BE), (e) hot water and alkali treated combed and GO modified jute/BE (HWACGO J/BE) and (f) hot water and alkali treated combed and GNP modified jute/BE (HWACGNP J/BE).

Supporting Information 2: Flexural properties of different jute/bio-epoxy composites

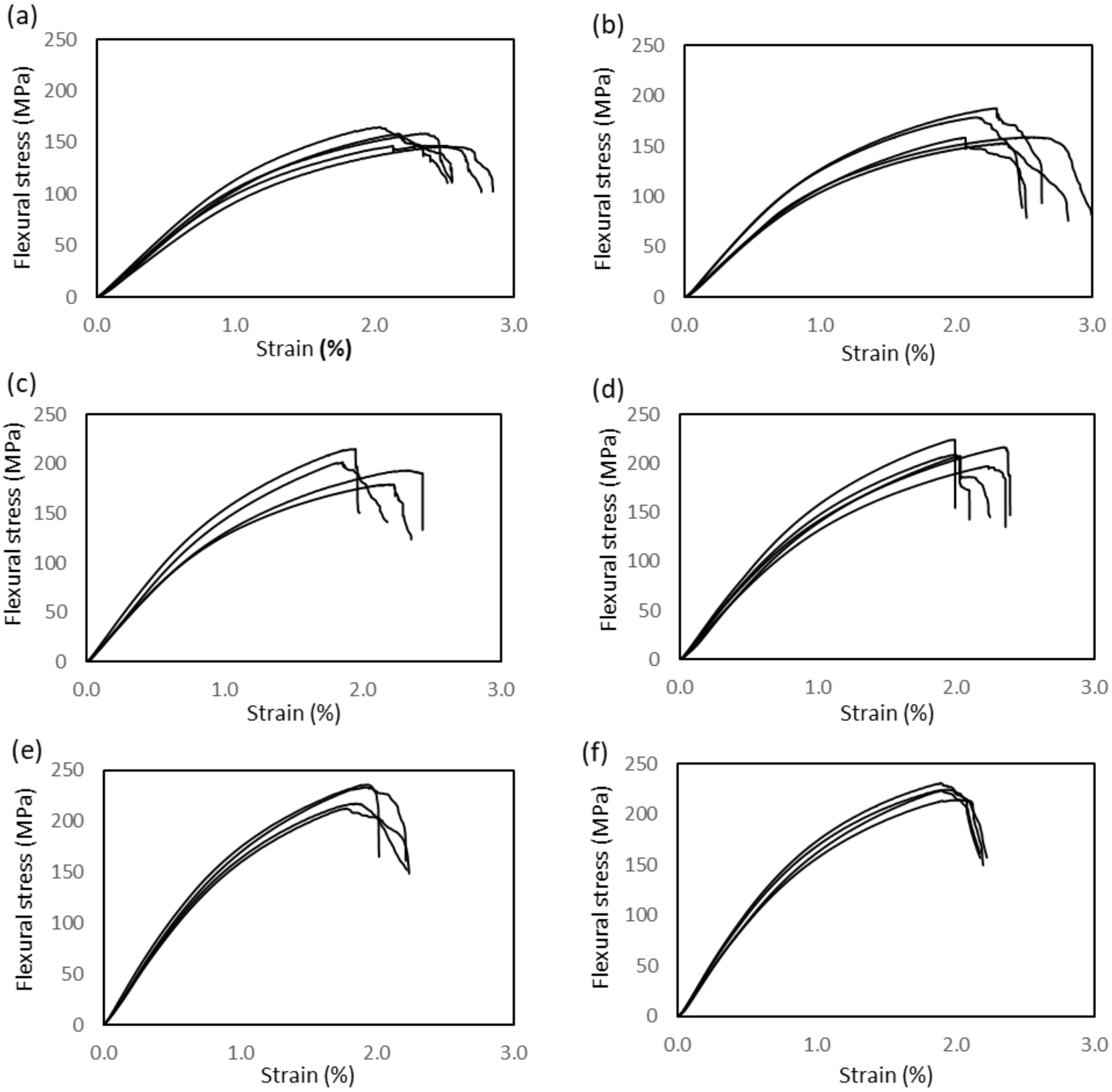

Figure S2: Flexural stress-strain graph of different jute/bio-epoxy (BE) composites; (a) untreated jute/BE (UT J/BE), (b) untreated combed jute/BE (UTC J/BE), (c) hot water treated combed jute/BE (HWC J/BE), (d) hot water and alkali treated combed jute/BE (HWAC J/BE), (e) hot water and alkali treated combed and GO modified jute/BE (HWACGO J/BE) and (f) hot water and alkali treated combed and GNP modified jute/BE (HWACGNP J/BE).

Supporting Information 3: Fabrication of jute/bio-epoxy composites

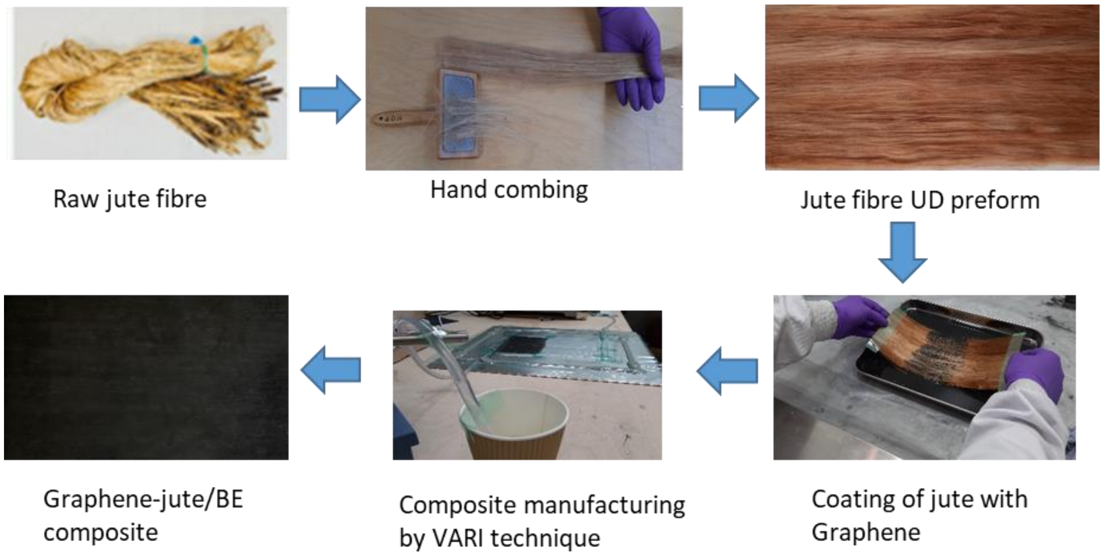

Figure S3: The flow diagram for the fabrication of jute/bio-epoxy composites.
